# Treatment of posttraumatic and focal osteoarthritic cartilage defects of the knee with autologous polymer-based three-dimensional chondrocyte grafts: 2-year clinical results

**DOI:** 10.1186/ar2180

**Published:** 2007-04-23

**Authors:** Christian Ossendorf, Christian Kaps, Peter C Kreuz, Gerd R Burmester, Michael Sittinger, Christoph Erggelet

**Affiliations:** 1 Department of Traumotology and Orthopaedic Surgery, University of Freiburg, Hugstetter Strasse 55, 79106 Freiburg, Germany; 2 Department of Rheumatology, Charité Campus Mitte, Charité – Universitätsmedizin Berlin, Charitéplatz 1, 10117 Berlin, Germany

## Abstract

Autologous chondrocyte implantation (ACI) is an effective clinical procedure for the regeneration of articular cartilage defects. BioSeed^®^-C is a second-generation ACI tissue engineering cartilage graft that is based on autologous chondrocytes embedded in a three-dimensional bioresorbable two-component gel-polymer scaffold. In the present prospective study, we evaluated the short-term to mid-term efficacy of BioSeed-C for the arthrotomic and arthroscopic treatment of posttraumatic and degenerative cartilage defects in a group of patients suffering from chronic posttraumatic and/or degenerative cartilage lesions of the knee. Clinical outcome was assessed in 40 patients with a 2-year clinical follow-up before implantation and at 3, 6, 12, and 24 months after implantation by using the modified Cincinnati Knee Rating System, the Lysholm score, the Knee injury and Osteoarthritis Outcome Score, and the current health assessment form (SF-36) of the International Knee Documentation Committee, as well as histological analysis of second-look biopsies. Significant improvement (*p *< 0.05) in the evaluated scores was observed at 1 and/or 2 years after implantation of BioSeed-C, and histological staining of the biopsies showed good integration of the graft and formation of a cartilaginous repair tissue. The Knee injury and Osteoarthritis Outcome Score showed significant improvement in the subclasses pain, other symptoms, and knee-related quality of life 2 years after implantation of BioSeed-C in focal osteoarthritic defects. The results suggest that implanting BioSeed-C is an effective treatment option for the regeneration of posttraumatic and/or osteoarthritic defects of the knee.

## Introduction

Cartilage has a low intrinsic regenerative and reparative capacity. Cartilage defects may be accompanied by pain, immobility, stiffness, and loss of quality of life, and can potentially lead to severe osteoarthritis in the long term. Because chondral lesions of the knee occur frequently and are a great health problem, several efforts were made to develop techniques for restoration of the cartilage surface and regeneration of the cartilage [1]. These common repair techniques comprise debridement, bone marrow-stimulating techniques, osteochondral grafting, and autologous chondrocyte implantation (ACI) [2-5]. Some of these techniques may be useful only for small defects [6], whereas others merely provide limited durability of the repair tissue [7,8]. Using the cell-based approach of ACI, such disadvantages were not reported [9,10].

Since the clinical introduction of ACI by Brittberg and colleagues [2], more than 15,000 patients worldwide have been treated with ACI [11] and a variety of clinical studies have documented the clinical effectiveness of implanting autologous culture-expanded chondrocytes for the regeneration of cartilage [9,12-14]. ACI involves the use of a periosteal flap or a collagen sheet [15], which is fixed to the surrounding cartilage and creates a reservoir for the injection of the autologous chondrocyte cell suspension. The use of ACI may therefore be delicate or even impossible in some regions of the knee. In ACI, the fixation of the periosteal flap or collagen sheets covering the chondrocyte suspension may be insecure, especially in degenerative defects lacking an intact cartilage rim. In addition, periosteal hypertrophy, ablation, uneven cell distribution, and loss of cells into the joint cavity may be potential sources of complications [16,17] resulting in repetition of surgery in up to 25 to 36% of the patients [15,18].

Recently, to overcome the intrinsic technical disadvantages of ACI, cartilage tissue engineering grafts were developed that use the regenerative potential of autologous chondrocytes with three-dimensional scaffolds to stabilize the graft. Meanwhile, clinical results show the safety and effectiveness of hyaluronan-based [19,20] and collagen-based autologous chondrocyte grafts for the repair of cartilage defects [21,22].

More advanced cartilage tissue engineering grafts ensure the even distribution of a high number of vital chondrocytes, mediate initial biomechanical stability, promote chondrocyte differentiation and the formation of cartilage matrix, inhibit chondrocyte proliferation, and allow easy handling of the graft by the surgeon [23]. The cartilage tissue engineering graft BioSeed^®^-C combines autologous chondrocytes with the tissue development-promoting properties of gel-like matrices in an initially mechanically stable bioresorbable polymer scaffold [24]. Polymer-based cartilage tissue engineering grafts for the regeneration of articular cartilage defects have been shown to facilitate development toward hyaline cartilage *in vitro *[25]. Three-dimensional assembly of chondrocytes in fibrin and polymer-based scaffolds initiates the redifferentiation of dedifferentiated culture-expanded chondrocytes, whereas matrix formation and tissue maturation occur *in vivo *after implantation of the graft [26]. Preclinical evaluation in the large-animal horse model showed the formation of a hyaline-like cartilage matrix as well as firm bonding of the graft to the adjacent healthy cartilage and to the subchondral bone tissue [27]. In BioSeed-C, the chondrocytes are immobilized in and protected by the fibrin-polymer matrix; additional cover materials or a healthy cartilage rim surrounding the defect are therefore not mandatory, and arthroscopical implantation and secure fixation are feasible [28].

The aim of this prospective study was to evaluate ACI using BioSeed-C, which is based on a bioresorbable two-component gel-polymer scaffold, for the treatment of posttraumatic, mild degenerative, and osteoarthritic defects of the knee. Magnetic resonance imaging (MRI) and histological analyses of the cartilage repair tissue as well as the clinical evaluation of a series of 40 patients with a 2-year clinical follow-up document the effectiveness of BioSeed-C for the treatment of cartilage defects.

## Materials and methods

### Patients

This ongoing prospective observational case report study was designed to investigate the effectiveness of BioSeed-C for the treatment of posttraumatic and degenerative cartilage defects of the knee. Candidates for inclusion were patients suffering from posttraumatic, mild degenerative, or osteoarthritic clinically significant, symptomatic defects of the articular cartilage of the knee. Patients gave their consent to participate.

From December 2001 to October 2002, 79 patients with chondral defects of the knee joint were treated with BioSeed-C. By November 2004, 40 out of 79 patients had reached a follow-up of 2 years. In this interim report, the clinical data of these 40 patients with 52 chondral defects and a minimum follow-up period of 2 years as available by November 2004 are presented. Patients' characteristics are given in Table 1.

**Table 1:** Patients' characteristics

Characteristic	Group 1 (Jaeger-Wirth score < 3)	Group 2 (Jaeger-Wirth score = 3)
Sex	13 female, 14 male	5 female, 8 male
Age (years)	34 (range 17–47)	38 (range 25–64)
Height (cm)	175 (range 160–189)	174 (range 164–181)
Weight (kg)	76.85 (range 54–100)	76.25 (range 60–102)
Body mass index	25 (range 19–34)	25 (range 21–31)
Defect size, 1st lesion (cm^2^)	4.2 (range 2–6)	5 (range 2–15)
Cartilage grade, 1st lesion, Outerbridge	IV (*n *= 27)	IV (*n *= 13)
Localization, 1st lesion	Medial femoral condyle (*n *= 19), lateral femoral condyle (*n *= 2), patella (*n *= 4), trochlea (*n *= 1), tibia (*n *= 1)	Medial femoral condyle (*n *= 8), lateral femoral condyle (*n *= 1), patella (*n *= 2), trochlea (*n *= 2)
2nd lesion	*n *= 6	*n *= 6
Defect size, 2nd lesion (cm^2^)	2.5 (range 1–4)	3 (range 2–4)
Cartilage grade, 2nd lesion, Outerbridge	IV (*n *= 6)	II (*n *= 1), IV (*n *= 5)
Localization, 2nd lesion	Patella (*n *= 4), trochlea (*n *= 2)	Medial femoral condyle (*n *= 2), lateral femoral condyle (*n *= 1), trochlea (*n *= 3),
Previous surgical procedures	High tibial osteotomy (*n *= 6), shaving (*n *= 15), abrasion arthroplasty (*n *= 4), microfracture/drilling (*n *= 8), ACI (*n *= 1), meniscectomy (*n *= 10), anterior cruciate ligament/collateral ligament reconstruction (*n *= 7), lateral release (*n *= 2)	High tibial osteotomy (*n *= 2), shaving (*n *= 8), abrasion arthroplasty (*n *= 4), microfracture/drilling (*n *= 4), ACI (*n *= 2), meniscectomy (*n *= 10), anterior cruciate ligament/collateral ligament reconstruction (*n *= 6)

ACI, autologous chondrocyte implantation.

In general, the average age of patients (18 females, 22 males; mean body mass index 25, range 19 to 34) was 36 years (range 17 to 64 years). The mean defect size of the first lesion was 4.6 cm^2 ^(range 2 to 15 cm^2^). All defects (first lesion) were classified as Outerbridge class IV [29]. The defects (first lesion) were situated on the medial femoral condyle (*n *= 27), on the lateral femoral condyle (*n *= 3), on the patella (*n *= 6), on the trochlea (*n *= 3), or on the tibia (*n *= 1). Previous surgical procedures were meniscectomies (*n *= 20), anterior cruciate ligament/collateral ligament reconstructions (*n *= 13), lateral releases (*n *= 2), abrasion arthroplasty (*n *= 7), drilling or microfracture (*n *= 13), shaving (*n *= 23), high tibial osteotomy (*n *= 8), or ACI (*n *= 3). When implanting BioSeed-C, 24 concomitant surgical procedures such as anterior cruciate ligament reconstruction (*n *= 10), high tibial osteotomy (*n *= 10), drilling/microfracture (*n *= 2), patella realignment surgery (*n *= 1), and medial capsular shift (*n *= 1) were performed.

To assess the degree of osteoarthritic degeneration of the defects, the Jaeger-Wirth score and the Kellgren-Lawrence score were applied. Thirteen of the patients had osteoarthritic cartilage defects showing a Jaeger-Wirth score of 3 [30,31]; 27 patients had posttraumatic and/or mild degenerative defects showing a Jaeger-Wirth score of 1 to 2, or had no signs of osteoarthritis.

Radiographs of the respective knee of 30 of the patients with osteoarthritic symptoms were taken preoperatively. Osteoarthritic degenerations were evaluated with the Kellgren-Lawrence scoring system [32] by two independent observers. The observer was blinded to the procedure. A Kellgren-Lawrence score of 2 or more defines osteoarthritis in a particular joint and was found in 22 patients with a clinical follow-up of 2 years.

Clinical examinations were performed at 3, 6, 12, and 24 months.

### Implantation of BioSeed-C

Autologous chondrocytes were isolated from approximately 250 mg of the patient's healthy cartilage that was harvested arthroscopically from a less weight-bearing area of the knee. For autologous chondrocyte cultivation, 100 ml of whole blood was collected with a conventional blood sampling system (Sarstedt AG, Nümbrecht, Germany). Chondrocytes were expanded *in vitro *and subsequently 20 million cells were rearranged three-dimensionally in fibrin and a polymer-based scaffold of polyglycolic/polylactic acid (polyglactin, vicryl) and polydioxanone. After careful debridement of the defective cartilage down to the subchondral bone, the tissue engineering graft (2 cm × 3 cm × 0.2 cm thick) was fitted to the size of the defect, implanted arthrotomically or arthroscopically at the defect site. The decision when to do arthroscopic implantation and when to perform open procedure was made on the basis of the location and size of the defects. Arthroscopic implantation was performed when defects were located on the medial/lateral condyle and when only one graft was sufficient to cover the defect.

As reported previously [28], for secure fixation the graft was armed on the corners with resorbable threads forming loops secured by three-fold knots that tightened pulley slings and served as anchors. On every corner of the defect, a k-wire was drilled transosseously with an inside-out technique. Then the pulley slings were pulled through the femoral bone by the guide wire and the knots were guided into the femoral bone, securely anchoring the graft.

### Follow-up treatment of patients after transplantation of BioSeed-C

On the day after surgery, the rehabilitation program started with continuous passive motion and subsequently allowed mobilization and partial loading with 15% of body weight as well as isometric tension exercises for 6 weeks. At 7 to 12 weeks after surgery, patients gradually increased the loading and performed specific strengthening exercises, active physiotherapy, and, if appropriate, ergometric training at a gentle level. Crutches were used to take weight off the operated knee. From week 13 onward, patients increased weight bearing and performed muscular and coordination exercises up to full weight bearing. Gentle exertion (such as cycling or jogging) was allowed after 6 months, and more strenuous activities and contact sports (such as tennis or football) after 12 months. The postoperative treatment plan was not mandatory and was drawn up specifically for each patient. Patient compliance was not monitored.

### Evaluation of clinical results

For evaluation of clinical results after transplantation of BioSeed-C, the modified Cincinnati Knee Rating System [33], the Lysholm score [34], the Knee injury and Osteoarthritis Outcome Score [35], and the International Knee Documentation Committee (IKDC) Knee Examination Form [36], with emphasis on the current health assessment form (SF-36), were applied and documented the clinical situation before transplantation of the graft and at 3, 6, 12, and 24 months after transplantation.

### Radiological and histological evaluation of cartilage repair

At 6 and 12 months after transplantation, repair and resurfacing of cartilage defects were evaluated by MRI (Philips Magnetron, Philips, Hamburg, Germany); 14 of the 79 patients had second-look arthroscopy for investigative and diagnostic purposes. After patient's consent had been obtained, biopsies of the repair tissue (*n *= 4) were harvested 9 to 12 months after transplantation of BioSeed-C, for investigative purposes. Paraffin sections were stained with alcian blue and nuclear fast red or with hematoxylin and eosin.

### Statistical analysis

For statistical analysis, the non-parametric Mann-Whitney rank sum test was applied; differences were considered significant at *p *< 0.05. All comparisons were performed between scorings at the individual points in time of the follow-up period against the preoperative scores.

## Results

### Postoperative radiological and histological evaluation of repair tissue formation

**Figure 1:**
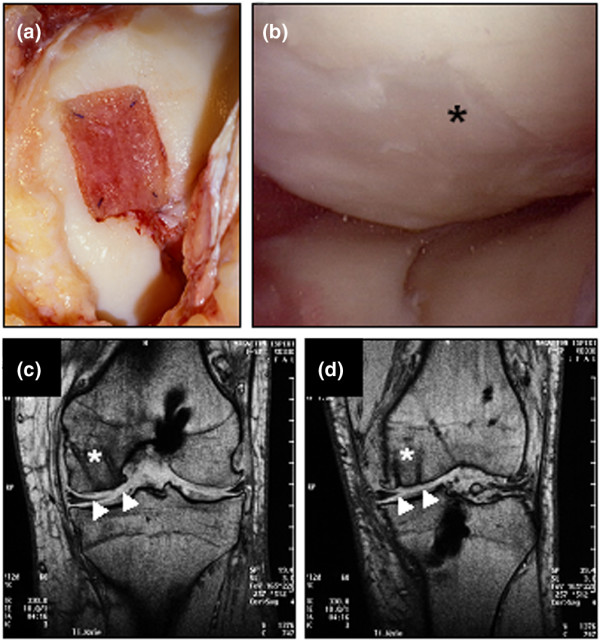
Arthroscopic and magnetic resonance imaging evaluation of cartilage defects treated with autologous chondrocyte grafts (BioSeed^®^-C). **(a) **Intra-operative situation of a cartilage defect situated at the femoral condyle covered with transosseously fixed BioSeed-C (20 mm × 30 mm). Note that the healthy cartilage rim is partly intact. **(b) **At 9 months after surgery, second-look arthroscopy showed the formation of a cartilage repair tissue of a tough condition (asterisk). Magnetic resonance imaging (MRI) at 6 months **(c) **and 12 months **(d) **after implantation of BioSeed-C shows transosseous drilling holes (white asterisks) due to fixation of the graft. The repair tissue covers the defect (white triangles) and gives a slightly altered MRI signal.

BioSeed-C was implanted arthrotomically or arthroscopically and fixed by transosseous anchor knots. Intra-operatively, no loosening, ablation, or derangement of the transplant occurred (Figure 1a). Postoperatively, no clinical signs of knee joint infection or persistent allergic reactions were evident. Neither knee joint extension deficiency nor flexion deficiency could be observed, and no knee joint effusion occurred.

Of the 79 patients, 14 underwent second-look arthroscopy as a result of symptoms such as persistent grinding, catching, pain, or swelling. The implanted grafts completely filled the defects and formed a tough hyaline-like cartilage (Figure 1b). MRI analysis at 6 months (Figure 1c) and 12 months (Figure 1d) after implantation showed good defect filling. The grafts were well integrated into the surrounding tissue and displayed good connection to the articular cartilage as well as to the underlying subchondral bone. No transplant loosening, debonding, ablation of the transplant, or articular constriction was observed. The newly formed cartilage showed a visible contrast in color to the surrounding cartilage, and transosseous drill holes were still evident. Subchondral edemas were not observed. Osseous healing after drilling did not differ from bone regeneration after osteosynthesis or reconstruction of the anterior cruciate ligament, for example.

Five patients underwent repeat surgery comprising synovectomies (*n *= 2), debridement (*n *= 1), total knee arthroplasty (*n *= 1), and removal of the graft (*n *= 1) in another hospital.

**Figure 2:**
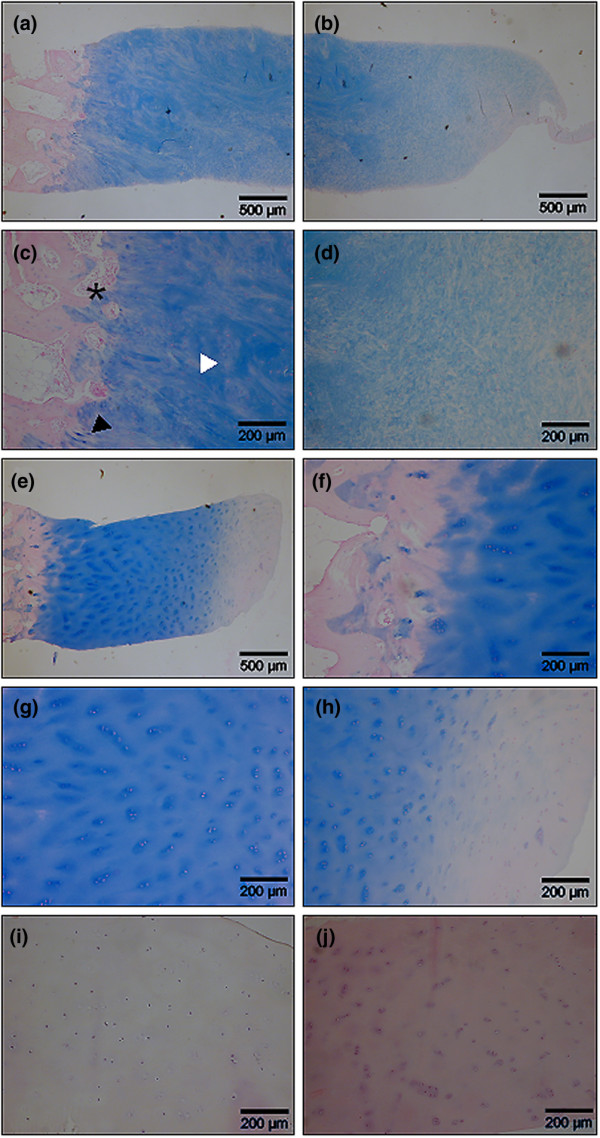
Histological analysis of second-look biopsy tissue from patients treated with BioSeed^®^-C. At 9 to 12 months after implantation, second-look biopsy tissue was stained for proteoglycans with alcian blue. One patient's biopsy tissue showed the formation of mixed repair tissue **(a-d) **with areas of fibrocartilage ((c), black triangle) and hyaline-like cartilage ((c), white triangle) and a firm bonding to the subchondral bone that was undergoing remodeling ((c), asterisk). Biopsy tissue from three patients **(e-j) **shows the formation of a hyaline-like cartilaginous repair tissue with intense staining of proteoglycans by alcian blue (e-h), good integration with the underlying bone tissue (f), viable, round cells within lacunae (g) and a smooth surface (h). Chondrocytes showed a columnar distribution and some clustering (g-j). Hematoxylin/eosin staining (i, j) of biopsy tissue of two patients confirmed the presence of viable chondrocytes and the absence of abnormal calcification, apoptosis, necrosis or formation of a fibrous repair tissue.

Histological analysis of biopsies after 9 to 12 months from implantation of BioSeed-C showed that one repair tissue appeared as a mixed tissue of hyaline-like and fibrous cartilage, whereas three biopsies documented the development toward a hyaline repair tissue (Figure 2). The mixed repair tissue (Figure 2a,b) showed good bonding of the engineered cartilage to the underlying bone with progressive remodeling of the subchondral bone tissue (asterisk in Figure 2c) and areas of fibrocartilage (black triangle in Figure 2c) and hyaline-like cartilage (white triangle in Figure 2c). The mixed repair tissue was rich in evenly distributed viable cells and was characterized by a proteoglycan-rich extracellular matrix (Figure 2d). Specimens with hyaline-like repair tissue (Figure 2e–j) showed intense staining of proteoglycans (Figure 2e) and good integration with the subchondral bone (Figure 2f). The chondrocytes were viable, round-shaped within lacunae, and showed a columnar distribution with some clustering (Figure 2g,i,j). The surface of the repair tissue appeared smooth and showed the typical articular cartilage surface-related tissue with a gradual decrease of proteoglycans within the extracellular matrix (Figure 2h). There were no signs of abnormal calcification or formation of a fibrous connective tissue within the repair tissue, and neither necrosis of the tissue nor apoptosis of chondrocytes was evident.

### Clinical evaluation of surgical results 2 years after transplantation of BioSeed-C

To assess the impact of osteoarthritic degeneration of the cartilage on the functional outcome, two subgroups were considered, namely those with a Jaeger-Wirth score of 3, representing patients with osteoarthritic degeneration, and those with a score less than 3, comprising patients with posttraumatic and mild degenerative defects.

**Figure 3:**
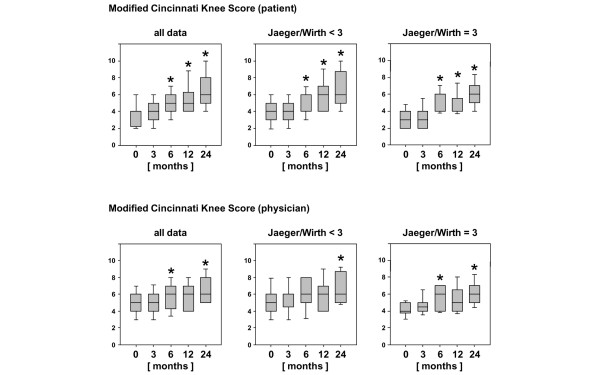
Clinical outcome after 2 years evaluated by the Modified Cincinnati Knee Rating System. The score from this system is shown for the entire patient cohort compared with patients with posttraumatic and mild degenerative defects (Jaeger-Wirth score < 3) and patients with osteoarthritic defects (Jaeger-Wirth score = 3). The preoperative and follow-up times are as indicated. Scores are presented as medians; the ends of the boxes define the 25th and 75th centiles, and error bars the 10th and 90th centiles. Where indicated (asterisks), differences were statistically significant (*p *< 0.05) compared with the preoperative situation.

According to the modified Cincinnati Knee Rating System (Figure 3), statistically significant improvements (*p *< 0.05) were observed as early as 6 months after implantation of BioSeed-C, independently of the degree of osteoarthritic degeneration of the cartilage. Rating by physicians yielded statistically significant improvement at 6 months and 2 years after implantation. Interestingly, the improvement at 6 months after implantation of BioSeed-C was observed only in patients with osteoarthritic degenerations. After 2 years, the median Cincinnati Knee Rating System score increased from 4.0 to 6.0 postoperatively.

**Figure 4:**
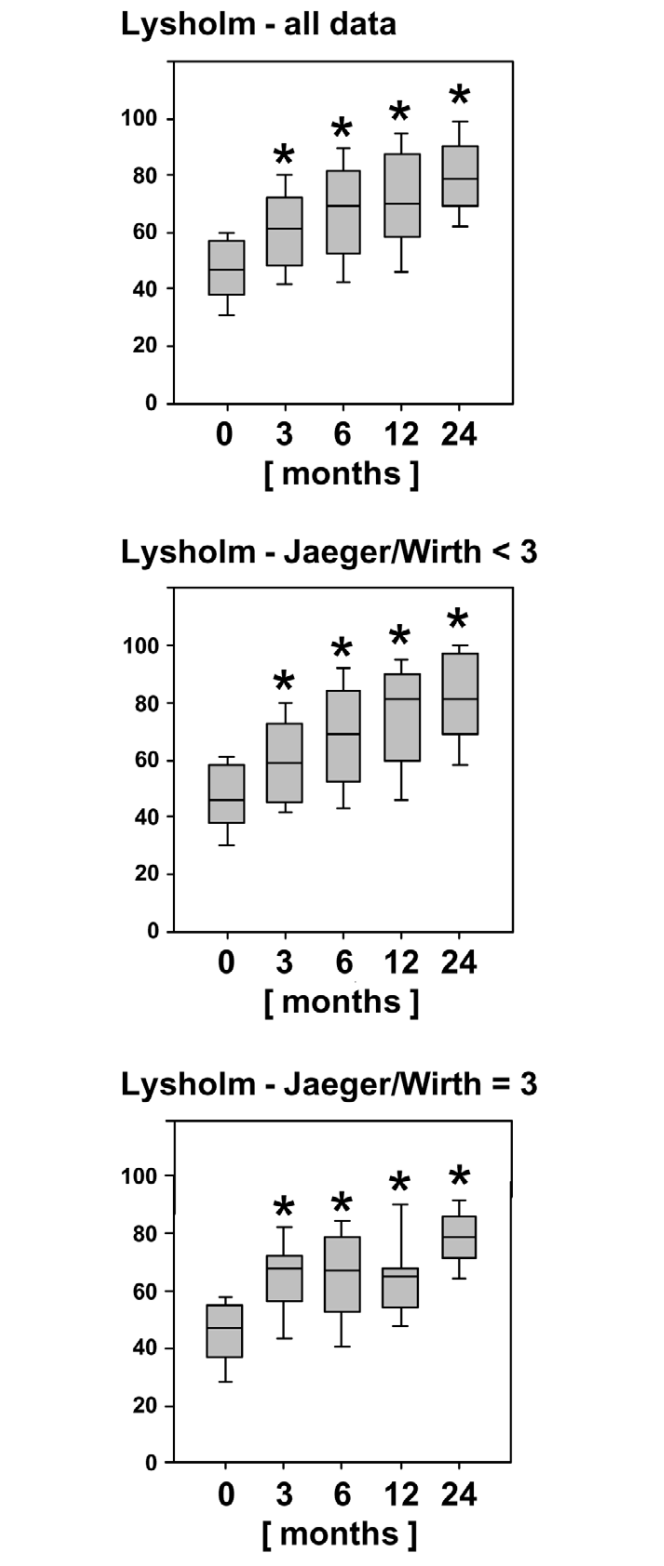
Clinical outcome after 2 years evaluated by the Lysholm score. The score is given for the entire patient cohort compared with patients with posttraumatic and mild degenerative defects (Jaeger-Wirth score < 3) and patients with osteoarthritic defects (Jaeger-Wirth score = 3). The preoperative and follow-up times are as indicated. Scores are presented as medians; the ends of the boxes define the 25th and 75th centiles, and error bars the 10th and 90th centiles. Where indicated (asterisks), differences were statistically significant (*p *< 0.007) compared with the preoperative situation.

In comparison with the preoperative scores, the Lysholm score (Figure 4) improved significantly (*p *< 0.007) in both groups of patients with osteoarthritic degeneration or with posttraumatic and/or mild degenerative defects as early as 3 months to up to 2 years after implantation of BioSeed-C. In comparison with the preoperative status, the median Lysholm score increased from 46.0 to 81.0 in patients with posttraumatic and/or mild degenerative defects and from 47.0 to 78.5 in patients with osteoarthritic degeneration, 2 years after implantation.

The Knee Injury and Osteoarthritis Outcome Score describes the patient's view about his knee and associated problems (Additional file 1). At 2 years of follow-up, the patient's status had improved significantly (*p *< 0.05) compared with the his or her preoperative situation, showing an increase in the mean scores of the subclasses pain (64.3 to 78.2), symptoms (68.2 to 78.9), activities of daily living (67.6 to 80.6), sports (25.8 to 45.7), and knee-related quality of life (26.9 to 52.9). Patients with posttraumatic and/or mild degenerative defects (Jaeger-Wirth score < 3) showed a significant improvement in only the subclass knee-related quality of life (28.4 to 52.9) 2 years after implantation, whereas patients with osteoarthritic degeneration showed improvement in pain (61.4 to 80.1), symptoms (64.0 to 78.4), and quality of life (23.6 to 52.8).

The health of patients was evaluated with the IKDC SF-36 current health assessment form (Additional file 2). Implantation of BioSeed-C resulted in a statistically significant (*p *< 0.05) increase in the mean scores after 6 months to 2 years in the subclasses physical functioning (42.8 to 64.6), role limitations due to physical health (25.7 to 53.6), bodily pain (38.9 to 61.6), general health problems (62.0 to 70.6), and social functioning (59.5 to 77.5) in all outcome measures compared with the patients' preoperative status. Evaluating the outcome measures according to the impact of the degree of osteoarthritic degeneration of the knee showed an improvement in the mean scores of the subclasses physical functioning (44.8 to 62.6), bodily pain (39.0 to 61.3), and social functioning (61.9 to 78.8) after implantation of BioSeed-C in posttraumatic and/or mild degenerative cartilage defects with a Jaeger-Wirth score of less than 3. Patients with osteoarthritic degeneration of the knee cartilage (Jaeger-Wirth score = 3) reported a significant impairment related to social functioning at 3 months after implantation of the cartilage transplant and showed a continual improvement in social functioning status from 6 months to 2 years. In addition, these patients showed a significant increase in the mean scores of the IKDC SF-36 subclasses physical functioning (38.8 to 68.3), role limitations due to physical health (10.4 to 52.1), and bodily pain (38.8 to 62.2) 2 years after implantation of BioSeed-C.

### Clinical evaluation of surgical results 2 years after implantation of BioSeed-C in defects of patients with radiographically confirmed osteoarthritis

**Figure 5:**
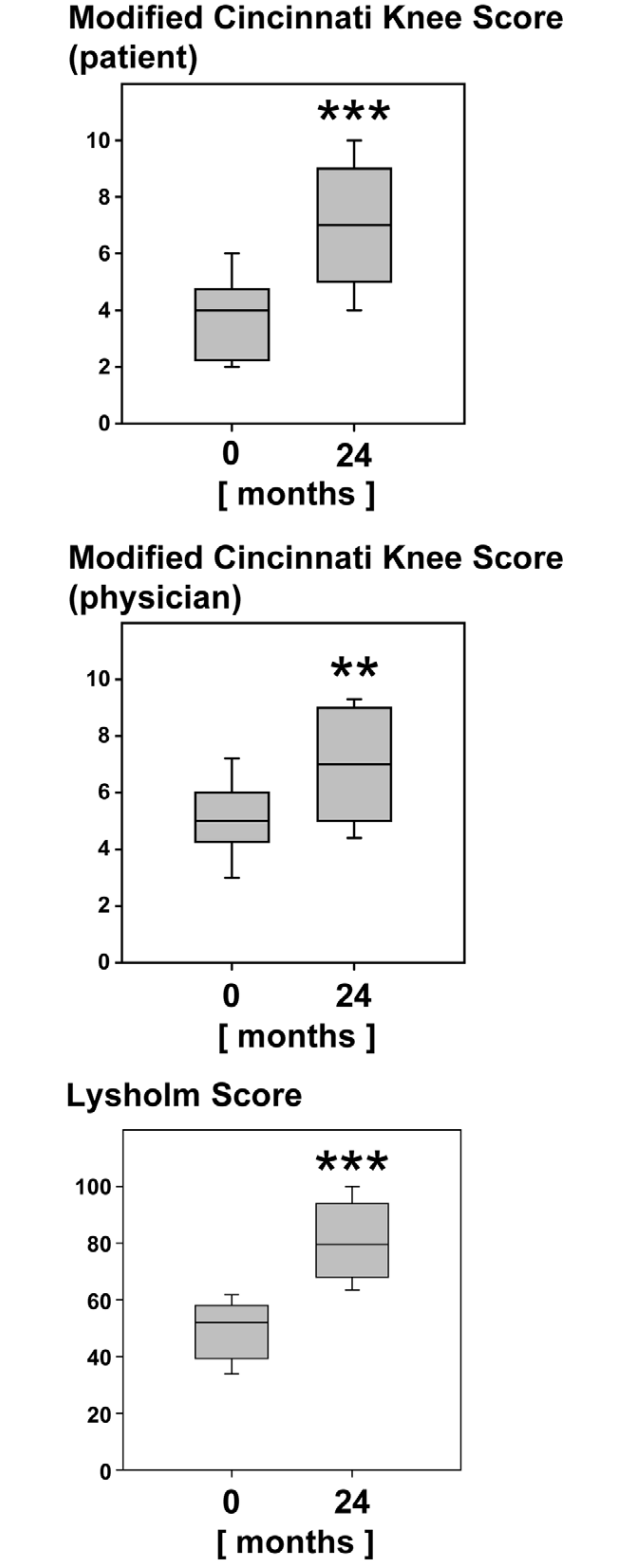
Clinical outcome after 2 years of implantation of BioSeed-C in osteoarthritic defects. The Modified Cincinnati Knee Rating System score and Lysholm score are given for 22 patients with radiologically confirmed osteoarthritic defects at 2 years after implantation of BioSeed-C. Osteoarthritis was defined according to a Kellgren-Lawrence score of 2 or more. Scores are presented as medians; the ends of the boxes define the 25th and 75th centiles, and error bars the 10th and 90th centiles. Where indicated (asterisks), differences were statistically significant (**p = 0.0074, ***p < 0.0001) compared with the preoperative situation.

Radiographs of the degenerated knee of 30 patients with osteoarthritic symptoms were taken preoperatively. Applying the Kellgren-Lawrence score showed that 22 of the 30 patients with a clinical follow-up of 2 years had osteoarthritis, having obtained a Kellgren-Lawrence score of 2 or more. The clinical outcome after 2 years after implantation of BioSeed-C in osteoarthritic focal defects was evaluated with the modified Cincinnati Knee Rating System and the Lysholm score (Figure 5). After 2 years, patients' (*p *< 0.0001) and physicians' (*p *= 0.0074) ratings showed a significant improvement in the median scores (patient 4.0 to 7.0, physician 5.0 to 7.0) of the modified Cincinnati Knee Rating System in comparison with the preoperative situation.

The Lysholm score showed significant improvement (*p *< 0.0001) in the median scores from 53.0 preoperatively to 79.5 after 2 years after implantation of BioSeed-C in focal osteoarthritic defects of the knee.

## Discussion

In the present study, we showed the benefit and reliability of the use of the autologous gel-polymer-based cartilage tissue engineering graft BioSeed-C for the treatment of full-thickness cartilage defects of the knee. The evaluation of the clinical outcome 2 years after implantation demonstrated that BioSeed-C is well suited for the treatment of patients with posttraumatic and mild degenerative defects as well as for the treatment of focal osteoarthritic defects.

The implantation of first-generation tissue engineering grafts such as ACI has been shown to be suitable for the regeneration of posttraumatic defects [12,37]. However, second-generation cartilage tissue engineering grafts using a variety of matrices to support the autologous chondrocytes were recently considered to be technically more attractive. For instance, Bartlett and colleagues reported the use of a collagen-based scaffold seeded with autologous chondrocytes for the treatment of 47 symptomatic chondral defects. After 1 year, the Cincinnati Knee Rating System score increased by 19.6, and 36.4% of the biopsies showed hyaline-like cartilage or a mixed repair tissue with fibrocartilage. Similar outcomes were obtained for defects treated with 'classical' ACI with a porcine-based collagen membrane covering the defects [21]. In a prospective study, 5 years after transplantation of cell-seeded collagen grafts, 8 of 11 patients rated the function of their knee better than before, and the clinical evaluation showed significant improvement in the Meyers score, the Lysholm-Gillquist score and the International Cartilage Repair Society score [22]. In a multicenter retrospective cohort study with Hyalograft C, a graft of autologous chondrocytes embedded in a derivative of hyaluronic acid, 91.5% of 141 patients with a follow-up from 2 to 5 years improved according to the IKDC subjective evaluation, and second-look biopsies showed hyaline-like cartilage [19]. The use of second-generation cartilage grafts based on collagen or hyaluronan matrices is therefore suggested to be as effective as ACI, both clinically and histologically.

Here we introduced the use of a new second-generation cartilage graft based on a biocompatible and bioresorbable two-component gel-polymer scaffold. The BioSeed-C concept of embedding autologous chondrocytes in a gel-like matrix distributed in a porous three-dimensional textile polymer structure goes back to more than 10 years of cartilage tissue engineering research [24,25]. Gel-like matrices such as fibrin allow the even distribution of a large number of vital chondrocytes within the graft and promote chondrocyte differentiation as well as the formation of a cartilaginous repair tissue, while the polymer scaffold mediates initial biomechanical stability and allows easy handling of the graft by the surgeon [23,38]. The arrangement of chondrocytes in three-dimensional scaffolds permits the arthroscopic implantation of cells, ensures secure fixation of the graft even in posttraumatic or degenerative defects without intact surrounding cartilage, and avoids the loss of cells into the joint cavity even after implantation in defects without an intact surrounding cartilage rim [28]. Drobnic and colleagues have shown that the transosseous fixation technique provides excellent stability of the polymer-based graft with high endpoint fixation strength and no detachment after continuous passive motion with loading in the initial postoperative period [39]. Mechanical testing of the scaffold used in this study showed that the graft withstands a maximal tensile load of up to 15 N when fixed transosseously or by chondral suture, whereas gel-like matrices or collagen membranes ruptured on being loaded with up to 10 N [40]. The capability of such polymer-based grafts to form an adequate cartilaginous repair tissue has been shown preclinically in several animal studies with cryopreserved and non-cryopreserved chondrocytes [41,42]. In addition, in a large-animal model system with Haflinger horses, polymer-based cartilage grafts have been shown to develop a cartilaginous repair tissue that is well integrated into the surrounding cartilage and is firmly bonded to the subchondral bone [27]. The bioresorbable scaffold material is composed of a copolymer of polyglactin (vicryl) and polydioxanone, shows good biocompatibility, and is frequently used clinically as suture material. In a rabbit dural tissue reaction study, the absorbable polyglactin and polydioxanone material guided tissue development with complete resolution of the inflammatory reaction during absorption and without any morphological sequelae [43]. Additionally, in cartilage regeneration, various *in vitro *and animal studies have shown that the scaffold supports cartilaginous tissue development with no signs of necrosis, apoptosis, or abnormal tissue reaction [26,27,38,44].

In this case series we demonstrated the benefit and reliability of the gel-polymer-based chondrocyte graft BioSeed-C for the treatment of posttraumatic and degenerative large full-thickness cartilage lesions of the knee. Histological analysis of the biopsies after implantation of BioSeed-C showed good formation of a cartilaginous repair tissue, and significant improvements in the clinical scores used could be ascertained, implying improvements in activities of daily living, ability to work, and in sports. However, despite these encouraging results one must take into account the fact that randomized clinical trials and long follow-up periods may offer more widespread information about the clinical effectiveness of a given cartilage repair approach [13,45-47]. ACI will therefore not be given an unrestricted recommendation for the treatment of full-thickness cartilage lesions of the knee. Nevertheless, patient status at 2 years of follow-up was reported as an important indicator for future outcome [10], because most of the complications of ACI occur during this period. In addition, major improvements in clinical scores, clinical evaluation, and subjective patient satisfaction were found during this time; for example, patients who did not return to sports within 2 years did not return later. The features identified as an indicator of a worse prognosis, namely multiple surgical procedures, higher age, and large defects, correspond to findings published by others [21].

With the gel-polymer-based three-dimensional cartilage grafts, 18% of the patients in this study underwent second-look arthroscopy as a result of grinding, catching, pain, or swelling of the knee. This is consistent with other studies reporting rates of revision surgery between 0% [48] and 25% [18]. Instead, 2 of 79 patients treated with BioSeed-C showed a failure of the graft, which represents a lower rate of graft failure than earlier findings, in which rates of failure in ACI with other implants between 5% [9] to 13% [18] were described. Repeat operations using the 'classical' ACI procedure as described by Peterson and Brittberg were mainly caused by problems associated with the periosteal flap [9,17,49]. This disadvantage of the original ACI technique could not occur in patients treated with BioSeed-C. Another advantage of the BioSeed technique is the reduced operating time. Furthermore, the procedure is less invasive because there is no need to harvest periosteum from the tibia. The complication rate is lower because there is no possibility of periosteal hypertrophy, which is a common complication of ACI [15]. Furthermore, the BioSeed-C procedure can be performed arthroscopically, which may be associated with faster recovery after surgery and with cosmetically better results. However, it should be taken into consideration that performing ACI arthroscopically is technically demanding and the use of specially designed instruments is essential.

After 2 years of follow-up, mean scores increased significantly, between 20 and 35% depending on the score analyzed. This indicates a significant decrease in the patient's pain and knee instabilities during activity. Intriguingly, Cincinnati score improvement at 6 months after implantation of BioSeed-C could be observed only in patients with osteoarthritic degenerations. In addition, patients suffering from osteoarthritic degenerations showed an improved Knee injury and Osteoarthritis Outcome Score in pain, symptoms, and quality of life, whereas scores for patients whose cartilage defects resulted from posttraumatic causes increased only in the quality of life section. According to the impact of the degree of osteoarthritic degeneration, patients with osteoarthritis of the knee reported impairment in four subclasses of the SF-36 score. Obviously, tissue regeneration, improvement in clinical scores, and improvement in patient's quality of life are achieved after implantation of polymer-based autologous cartilage grafts even in osteoarthritic conditions.

Currently, ACI is considered not to be indicated for osteoarthritic patients. In spite of this, many young patients suffer from early stages of osteoarthritis or display deformities predisposing to osteoarthritis that are idiopathic or follow trauma. These patients lack good treatment options and are too young for total joint replacement. This is particularly true for those having an active lifestyle that includes sports or demanding recreational activities. Most of the patients of the present study suffered preoperatively from pain or dysfunction of the knee joint. They frequently underwent several failed cartilage repair procedures, and subsequently had to endure massive restrictions of quality of life, ability to work, and sporting activities. Thus, we consider the outcome of this study as a promising result for the treatment of large cartilage lesions of the knee, particularly for this challenging patient cohort with difficult cartilage conditions and in need of a variety of concomitant surgery procedures such as anterior cruciate ligament reconstruction or high tibial osteotomy. Besides, as a first step, it would be a beneficial effort to postpone total joint replacement for a decade. Recently, the effectiveness of second-generation cartilage grafts has been shown for the treatment of osteoarthritic knees. Hollander and colleagues reported the use of a hyaluronan-based second-generation cartilage tissue engineering graft for the treatment of osteoarthritic knees [50]. Histological and biochemical analyses of second-look biopsies documented the regeneration of cartilage as early as about 1 year after transplantation in 10 of 23 patients and showed that osteoarthritis did not inhibit the regeneration progress.

## Conclusion

The present study supports the use of the three-dimensional autologous cartilage graft, BioSeed-C, for the treatment of posttraumatic and osteoarthritic cartilage defects of the knee. Clinical evaluation 2 years after implantation showed that the treatment of posttraumatic and osteoarthritic defects with BioSeed-C leads to an improvement in the patient's condition as documented by the significant improvement in reliable clinical outcome scores. Further long-term studies with more patients are needed to prove the effectiveness of tissue engineering cartilage grafts to postpone total joint replacement in osteoarthritis.

## Abbreviations

ACI = autologous chondrocyte implantation; IKDC = International Knee Documentation Committee.

## Competing interests

CK is an employee of TransTissue Technologies GmbH. TransTissue Technologies GmbH is a subsidiary of BioTissue Technologies GmbH, which produces and distributes BioSeed^®^-C. MS works as a consultant for TransTissue Technologies GmbH. CE works as a consultant for BioTissue Technologies GmbH. All other authors declare that they have no competing interests.

## Authors' contributions

CO and CK performed the data evaluation and drafted the manuscript. PCK participated in the patient data collection. GRB and MS partly conceived the study and participated in the study design. CE conceived the study, participated in its design and coordination, performed the surgical procedures, and was involved in the patient data collection and interpretation. All authors read and approved the final manuscript.

## Supplementary Material

Additional file 1 An EPS file showing the clinical outcome after 2 years evaluated by the Knee injury and Osteoarthritis Outcome Score (KOOS). Click here for file

Additional file 2 An EPS file showing the clinical outcome after 2 years, evaluated by the International Knee Documentation Committee (IKDC) SF-36 score. Click here for file
